# From the dual function lead AP2238 to AP2469, a multi-target-directed ligand for the treatment of Alzheimer's disease

**DOI:** 10.1002/prp2.23

**Published:** 2014-03-24

**Authors:** Andrea Tarozzi, Manuela Bartolini, Lorna Piazzi, Luca Valgimigli, Riccardo Amorati, Cecilia Bolondi, Alice Djemil, Francesca Mancini, Vincenza Andrisano, Angela Rampa

**Affiliations:** 1 Department for Life Quality Studies, Alma Mater Studiorum-University of BolognaCorso d'Augusto 237, 47921, Rimini, Italy; 2 Department of Pharmacy and Biotechnology, Alma Mater Studiorum-University of BolognaVia Belmeloro 6, 40126, Bologna, Italy; 3 Department of Chemistry “Giacomo Ciamician”, Alma Mater Studiorum-University of BolognaVia S. Giacomo 11, 40126, Bologna, Italy; 4 ICIQ - Institute of Chemical Research of CataloniaAvenida Països Catalans 16, 43007, Tarragona, Spain

**Keywords:** Alzheimer's disease, *β*-amyloid peptide, neuroprotective properties, anti-inflammatory properties, multi-target ligand, catechol derivative

## Abstract

The development of drugs with different pharmacological properties appears to be an innovative therapeutic approach for Alzheimer's disease. In this article, we describe a simple structural modification of AP2238, a first dual function lead, in particular the introduction of the catechol moiety performed in order to search for multi-target ligands. The new compound AP2469 retains anti-acetylcholinesterase (AChE) and beta-site amyloid precursor protein cleaving enzyme (BACE)1 activities compared to the reference, and is also able to inhibit A*β*_42_ self-aggregation, A*β*_42_ oligomer-binding to cell membrane and subsequently reactive oxygen species formation in both neuronal and microglial cells. The ability of AP2469 to interfere with A*β*_42_ oligomer-binding to neuron and microglial cell membrane gives this molecule both neuroprotective and anti-inflammatory properties. These findings, together with its strong chain-breaking antioxidant performance, make AP2469 a potential drug able to modify the course of the disease.

## Introduction

Alzheimer's disease (AD) is a progressive neurodegenerative disorder of the brain associated with cognitive impairment, memory loss, and changes in personality and behavior, affecting a large portion of the aging population. Considerable research is in progress to understand the pathogenesis of the disease and find a cure.

Structural modifications in the brain have been identified as pathological hallmarks of AD, especially in regions such as the cortex, basal forebrain nuclei, and hippocampus (Selkoe 1993): synaptic loss, with drastic reduction in the cholinergic tone, extracellular senile plaque, mostly composed of aggregated *β*-amyloid peptide (A*β*) and intracellular neurofibrillary tangles, containing hyperphosphorylated Tau protein (Kowall 1999; Fine, 1999).

Recent experimental evidence has demonstrated that neuronal exposure to very low concentrations of soluble A*β* oligomers can initiate neurophysiological changes which are likely related to the synaptic dysfunction associated with AD. A*β* is a proteolytic fragment derived from the amyloid precursor protein, APP, which is processed by the enzyme *α*-secretase, in physiological conditions, to generate small and soluble peptides (Selkoe 1994; Hardy and Selkoe 2002). In the AD affected brain a second pathway, known as the “amyloidogenic pathway”, is activated, involving the sequential action of *β*-secretase (or beta-site amyloid precursor protein cleavage enzyme [BACE1]) and γ-secretase, to generate two predominant A*β* peptides, either 40 or 42 amino acids in length, that are able to aggregate into fibrils via soluble oligomers, leading, as generally accepted, to neuronal toxicity. The therapeutic approach should therefore try to decrease amyloid production (Citron 2002; Guo and Hobbs 2006; Hills and Vacca 2007), or block accumulation of misfolded peptide aggregates (Talaga 2001; Estrada and Soto 2007).

Among the mechanisms involved in A*β*-mediated neurotoxicity, oxidative stress has been recognized as an early event that plays a crucial role in the pathogenesis of AD. Several studies showed that A*β* generates various reactive oxygen species (ROS), such as hydrogen peroxide, hydroxyl radical, and superoxide anion by directly interacting with metals or indirectly by impairment of mitochondrial activity (Bobba et al. 2010). In addition, the overload of ROS induces accumulation of A*β* establishing a vicious circle that reinforces the oxidative stress with strengthening of oxidative damage at neuronal level (Tamagno et al. 2008).

Among the mechanisms involved in neuronal dysfunction and death, the accumulation of A*β* peptide, in different aggregation forms, including soluble oligomers and insoluble fibrils, has also been linked to inflammation responses in AD (Glass et al. 2010). It is recognized that the microglial cells enhance and amplify neuronal damage induced by A*β*. It seems that this phenomenon, in turn, induces more widespread damage – called reactive gliosis – to neighboring neurons, resulting in a perpetuating cycle of neuron death (Block et al. 2007). A*β* has been shown to activate microglial cells, in part by signaling through toll-like receptors and glycosylation end products, which in turn induce the production of factors such as nitric oxide (NO), ROS, proinflammatory cytokines, chemokines and prostaglandins that promote neuronal death (Glass et al. 2010).

Due to the complexity of this disease and the involvement of different proteins in its progression, the modulation of a single factor might not be sufficient to produce the desired efficacy. Indeed, the current management of AD is being reviewed and researchers are now turning to the design of structures that could be able to simultaneously interact with different targets involved in the pathogenic process.

Our research group has been involved for several years in the development of potential drugs for AD. In particular, AP2238 was the first dual binding site human acetylcholinesterase (hAChE) inhibitor (Piazzi et al. 2003) for which the simultaneous inhibition of the catalytic activity and the proaggregatory action of AChE on amyloid-*β* peptides was verified. Extensive structure–activity relationship studies (Piazzi et al. 2007) have shown that the structure of AP2238 is crucial for optimal activity. Indeed, only the introduction of an ethyl group (AP2243) instead of a methyl group on the basic nitrogen led to an improvement in the anti-AChE activity without decreasing the inhibitory potency on the AChE-induced A*β* aggregation.

**Figure 1:**
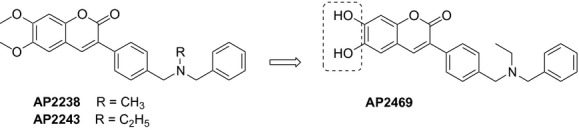
Drug design and synthesis of AP2469.

In this article we describe a simple structural modification of AP2243 (Fig. 1), leading to the introduction of the catechol moiety. This structural modification was based on the observation that catechol itself and catechol derivatives such as dopamine and quercetin were recently shown to possess antiaggregating properties (Di Giovanni et al. 2010; Huong et al. 2010). In addition, quercetin was also shown to inhibit BACE1 in both a cell-free system and in neuronal cells (Shimmyo et al. 2008). Finally, it is also well-known that catechols have antioxidant activity, which might be beneficial in the treatment of AD patients (Amorati and Valgimigli 2012; Valgimigli and Pratt 2012). Therefore, the simple switch from the 6,7-methoxy-2H-2-chromenone nucleus of AP2238 and AP2243 to a catecholic one is expected to enlarge the neuroprotective profile of the resulting compound and obtain an effective multi-target directed ligand. In this regard, we evaluated the neuroprotective profile of AP2238 and AP2243 in terms of anticholinesterase and antiaggregating activities, BACE1 inhibition, together with antioxidant, neuroprotective, anti-inflammatory activity at neuronal and microglial cell level.

## Materials and Methods

### Chemistry

#### General methods

Melting points were measured in glass capillary tubes on a Büchi SMP-20 apparatus (Milan, Italy) and are uncorrected. Direct infusion ES-MS spectra were recorded on a Waters Micromass ZQ 4000 apparatus (Milan, Italy). ^1^H NMR experiments were recorded on Varian VXR 300 MHz instruments (Palo Alto, CA). Chemical shifts are reported in parts per million (ppm) relative to tetramethylsilane, and spin multiplicities are given as s (singlet), d (doublet), t (triplet), dd (double doublet), dt (double triplet), m (multiplet) or br (broad). The results of the elemental analysis are within 0.4% of the theoretical values. Chromatographic separations were performed on silica gel columns (Kieselgel 40, 0.040–0.063 mm; Merck, Darmstadt, Germany) by flash chromatography. Compounds were named following IUPAC rules as applied by Beilstein-Institut AutoNom, version 2.1 (Frankfurt, Germany), a PC integrated software package for systematic names in organic chemistry. 3-{4-[(Benzylethylamino)-methyl]phenyl}-6,7-dihydroxychromen-2-one (AP2469). A mixture of AP2243 (Piazzi et al. 2007) (0.5 g) and 48% HBr (10 mL) was heated to reflux for 3 h and then poured onto ice. The aqueous layer was basified with NaHCO_3_, and extracted with CH_2_Cl_2_. The combined organic layers were dried and concentrated under reduced pressure to give a residue that was converted in hydrochloride salt and crystallized from MeOH/Et_2_O. AP2469 (55% yield) was obtained as a solid: mp 198–199. ^1^H NMR: δ 1.08 (t, 3H), 2.49–2.51 (m, 2H), 3.59 (s, 2H), 3.61 (s, 2H), 6.83 (s, 1H), 7.12–7.46 (m, 8H), 7.70 (d, 2H, *J* = 8.1 Hz), 7.95 (s, 1H). ES-MS (base) *m/e*: 402 (M + 1). Anal. (C_25_H_24_ClNO_4_), Calc.: C 68,57; H 5,52; N, 3,20. Found: C 68,56; H 5,53; N, 3,23.

### Inhibition of AChE and butyrylcholinesterase

The method of Ellman et al. (1961) was followed. Five different concentrations of tested compounds were selected in order to obtain inhibition of AChE or butyrylcholinesterase (BuChE) activity comprised between 20% and 80%. In detail, the following ranges of concentrations were screened: AP2238, 6–320 nmol/L and 11600–116000 nmol/L for AChE and BuChE, respectively; AP2243, 3–230 nmol/L and 3.4–230 μmol/L for AChE and BuChE, respectively; AP2469, 2.60–29.0 μmol/L and 15.5–125 μmol/L for AChE and BuChE, respectively; galanthamine 0.440–8.80 μmol/L 00 nmol/L and 4.50–91.50 μmol/L for AChE and BuChE, respectively. The assay solution consisted of a 0.1 mol/L potassium phosphate buffer pH 8.0, with the addition of 340 μmol/L 5,5′-dithio-bis(2-nitrobenzoic acid), 0.02 unit/mL of human recombinant AChE or BuChE derived from human serum (Sigma Chemical, Milan, Italy), and 550 μmol/L of substrate (acetylthiocholine iodide or butyrylthiocholine iodide, respectively). Compounds were added to the assay solution and preincubated at 37°C with the enzyme for 20 min before the addition of substrate. The enzyme activity was determined by monitoring the increase in absorbance at 412 nm over time. Assays were carried out with a blank containing all components except AChE or BuChE in order to account for nonenzymatic reaction. The reaction rates were compared, and the percent inhibition due to the presence of test compounds was calculated. Each concentration was analyzed in triplicate, and IC_50_ values were determined graphically from inhibition curves (log inhibitor concentration vs. percent inhibition).

### Inhibition of A*β*_42_ self-aggregation

Antiaggregating properties of compounds AP2469 were investigated in vitro by a thioflavin T (ThT)-based assay. 1,1,1,3,3,3-hexafluoro-2-propanol pretreated A*β*_1-42_ samples (Bachem AG, Switzerland) were resolubilized with a CH_3_CN/0.3 mmol/L Na_2_CO_3_/250 mmol/L NaOH (48.4/48.4/3.2) mixture to have a stable stock solution ([A*β*_1-42_] = 500 μmol/L) (Bartolini et al. 2007). Experiments were performed by incubating the peptide in 10 mmol/L phosphate buffer (pH = 8.0) containing 10 mmol/L NaCl, at 30°C for 24 h (final A*β* concentration = 50 μmol/L) with and without inhibitor. Volumes of 1.5 mmol/L stock solutions were prepared by dissolving tested inhibitors in methanol (c = 3.0 mmol/L); dilutions were prepared in the assay buffer in order to achieve concentrations ranging from 5 to 50 μmol/L in the final assay. Blanks containing tested inhibitors and ThT were also prepared and evaluated to account for quenching and interference related to inhibitor fluorescence. To quantify amyloid fibril formation, the ThT fluorescence method was used (Naiki et al. 1991; LeVine 1993). After incubation, samples were diluted to a final volume of 2.0 mL with 50 mmol/L glycine-NaOH buffer (pH 8.5) containing 1.5 μmol/L ThT. A 300-sec-time scan of fluorescence intensity was carried out (λ_exc_ = 446 nm; λ_em_ = 490 nm), and values at plateau were averaged after subtracting the background fluorescence of 1.5 μmol/L ThT solution and of AP2469. The fluorescence intensities in the absence and in the presence of the inhibitor were compared and the percent inhibition was calculated.

### Inhibition of BACE 1

AP2469 was evaluated for its ability to inhibit BACE1, using a fluorescence resonance energy transfer assay. Purified Baculovirus-expressed BACE 1 (*β*-secretase) in 50 mmol/L Tris (pH = 7.5), 10% glycerol, and rhodamine-derivative substrate (Panvera peptide) were purchased from Invitrogen (Milan, Italy). Sodium acetate and dimethyl sulfoxide (DMSO) were from Sigma Aldrich. Purified water from Milli-RX system (Millipore, Milford, MA) was used to prepare buffers and standard solutions. Spectrofluorometric analyses were carried out on a Fluoroskan Ascent multiwell spectrofluorometer (*λ*_exc_ = 544 nm; *λ*_em_ = 590 nm) by using black microwell (96 wells) Corning plates (Sigma Aldrich, Milan, Italy).

Stock solutions of the tested compounds were prepared in DMSO and diluted with 50 mmol/L sodium acetate buffer pH = 4.5. Specifically, 20 μL of BACE1 enzyme (12.8 nmol/L, final concentration) were incubated with 20 μL of test compound for 60 min. To start the reaction, 20 μL of Panvera peptide (0.25 μmol/L, final concentration) was added to each well. The mixture was incubated at 37°C for 60 min. To stop the reaction, 20 μL of BACE1 stop solution (sodium acetate 2.5 mol/L) was added to each well. The spectrofluorometric assay was then performed by reading the fluorescence signal at 590 nm.

The DMSO concentration in the final mixture maintained below 5% (v/v) guaranteed no significant loss of enzyme activity. The fluorescence intensities with and without inhibitor were compared and the percent inhibition due to the presence of test compounds was calculated. The background signal was measured in control wells containing all the reagents, except BACE 1 and subtracted. The % inhibition due to the presence of increasing test compound concentration was calculated by the following expression: 100 − (IF_i_/IF_o_ × 100) where IF_i_ and IF_o_ are the fluorescence intensities obtained for BACE 1 in the presence and in the absence of inhibitor, respectively. The inhibition curve was obtained by plotting the % inhibition versus the logarithm of inhibitor concentration in the assay sample, when possible. The linear regression parameters were determined and the IC_50_ extrapolated (GraphPad Prism 4.0, GraphPad Software Inc.).

To demonstrate inhibition of BACE1 activity, a statine peptide (H-Lys-Thr-Glu-Glu-Ile-Ser-Glu-Val-Asn-[Statine(3S,4S)]-Val-Ala-Glu-Phe-OH) derivative (Merck) was serially diluted into the reaction wells (IC_50_ = 0.018 μmol/L).

### Determination of neuroprotective and anti-inflammatory effects

#### A*β*_1-42_ oligomers and A*β*_25-35_ peptide preparation for cytotoxicity assay

A*β*_1-42_ and A*β*_25-35_ peptides (Bachem AG) of the same lot were first dissolved in hexafluoroisopropanol to 1 mg mL^−1^, sonicated, incubated at room temperature for 24 h and lyophilized. The resulting unaggregated A*β*_1-42_ and A*β*_25-35_ peptide film was dissolved with dimethylsulfoxide and stored at −20°C until use. The A*β*_1-42_ aggregation to oligomeric form was prepared as previously described (Maezawa et al. 2006). The morphology of oligomeric A*β*_1-42_ forms obtained was checked using transmission electron microscopy as previously reported (Tarozzi et al. 2010a,b).

#### Cell cultures

Human neuronal (SH-SY5Y) and monocytic (THP-1) cells were routinely grown in Dulbecco's modified Eagle' medium and Royal Park Memorial Institute medium, respectively, supplemented with 10% fetal bovine serum, 2 mmol/L glutamine, 50 U mL^−1^ penicillin, and 50 μg mL^−1^ streptomycin at 37°C in a humidified incubator with 5% CO_2_. THP-1 cells are transformed human mononuclear cells that have a range of properties similar to microglial cells and represent a suitable cellular model for anti-inflammatory effects (Klegeris and McGeer 2000; Yates et al. 2000).

#### Determination of A*β*_1-42_ oligomer and A*β*_25-35_ peptide-induced cytotoxicity

To evaluate the protective effects of compounds against A*β*_1-42_ oligomer- and A*β*_25-35_ peptide-induced cytotoxicity, the SH-SY5Y and THP-1cells were seeded in 96-well plates at 3 × 10^4^ cells/well and 5 × 10^4^ cells/well, respectively, incubated for 24 h and subsequently treated with 5 μmol/L of A*β*_1-42_ oligomers and A*β*_25-35_ peptide for 3 h at 37°C in 5% CO_2_, in the presence or absence of various concentrations of compounds (1–30 μmol/L). The cell viability in terms of mitochondrial metabolic function was evaluated by the reductionin 3-(4,5-dimethyl-2-thiazolyl)-2,5-diphenyl-2H-tetrazolium bromide (MTT) to formazan as previously described (Tarozzi et al. 2010a,b). The cellular reduction in MTT represents an indicator of the initial events underlying the mechanism of A*β*_1-42_ oligomer and A*β*_25-35_ peptide cytotoxicity. Briefly, the treatment medium was replaced with MTT (5 mg/mL) in phosphate-buffered saline (PBS) for 2 h at 37°C in 5% CO_2_. After washing with PBS, the formazan crystals were dissolved with isopropanol. The amount of formazan was measured (570 nm, ref. 690 nm) using a spectrophotometer (TECAN®, GENios, Salzburg, Austria). The neuronal viability is expressed as a percentage of control cells.

#### Determination of A*β*_1-42_ oligomer binding to the cell surface

SH-SY5Y and THP-1 cells were seeded in 96-well plates at 5 × 10^3^ cells/well and 5 × 10^4^ cells/well, respectively, for 24 h. At the end of incubation, the medium was changed with a fresh one with A*β*_1-42_ oligomer (5 μmol/L) and various compounds at 30 μmol/L for 30 min and then washed twice with PBS. The residual A*β*_1-42_ oligomer cell complex was stained with 1 μmol/L of Congo red (CR) in PBS for 20 min and measured with a spectrophotometer (TECAN®, GENios) at 540 nm (bound CR). CR values are reported as percent increases in treated cells versus untreated cells.

### Determination of antioxidant activity

#### Determination of intrinsic antioxidant activity by electron paramagnetic resonance spectroscopy

The reaction between AP2469 or its methylated analogue and peroxyl radical (*ROO* · ) was investigated by measuring the kinetics of oxygen consumption during the azobisisobutyronitrile (AIBN)-initiated autoxidation of styrene in chlorobenzene, whichs proceed via a well-established radical-chain mechanism reported in equations 1–4 (Burton et al. 1985). In the presence of an antioxidant, peroxyl radicals are trapped in reactions 5–6, the efficiency of this process being dependent on the rate constant of the reaction 5, *k*_inh_.

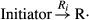
(1)

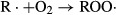
(2)

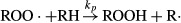
(3)

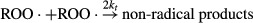
(4)

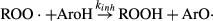
(5)

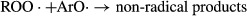
(6)

During the inhibition period, the oxygen consumption is given by equation 7, where *k*_p_ is the propagation rate constant of the oxidizable substrate (41 mol/L^−1^s^−1^ for styrene) (Burton et al. 1985; Amorati et al. 2010, 2011) and τ the length of the induction period. The number of peroxyl radicals trapped by each antioxidant molecule, *n*, was experimentally determined from equation 8, where *R*_i_ is the rate of free radical initiation obtained using the *α*-tocopherol analogue 2,2,5,7,8-pentamethyl-6-chromanol (PMHC) as reference antioxidant for which *n* = 2.^i^

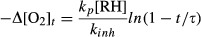
(7)

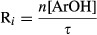
(8)

Autoxidation experiments were performed in a two-channel oxygen-uptake apparatus, based on a Validyne DP 15 differential pressure transducer, already described elsewhere (Amorati et al. 2001). The entire apparatus was immersed in a thermostated bath to ensure a constant temperature within ±0.1°C. In a typical experiment, an air-saturated solution of styrene or cumene in chlorobenzene containing AIBN (5 × 10^−2^ mol/L) was equilibrated with the reference solution containing an excess of PMHC (1 × 10^−3^) in the same solvents at 30°C until steady oxygen consumption was detected. A small amount of a concentrated solution of antioxidant (about 1 × 10^−3^ mol/L) was injected into both the reference and sample flasks, and the oxygen consumption in the sample was measured, after calibration of the apparatus, from the differential pressure recorded with time between the two channels.

#### Determination of A*β*_1-42_ oligomer-induced intracellular ROS formation

The intracellular hydrogen peroxide and superoxide anion formation induced by A*β*_1-42_ oligomer at SH-SY5Y cell level was determined using, respectively, the probe 2′,7′-dichlorodihydrofluorescein diacetate (DCFH-DA, λ_exc_ = 485 nm, λ_em_ = 535 nm), and dihydroethidium (DHE, λ_exc_ = 380 nm, λ_em_ = 445 nm). SH-SY5Y cells were cultured in BD Falcon™ 8-well Culture slides (surface area 0.7 cm^2^/well) at 1 × 10^4^ cells/well for 24 h. The cells were then treated for 3 h with various concentrations of compounds (3–30 μmol/L) at 37°C prior to the treatment of 3 h with A*β*_1-42_ oligomer (5 μmol/L). At the end of treatment, the cells were washed and incubated with DCFH-DA (5 μmol/L) or DHE (10 μmol/L) for 30 min in the dark. After removal of the probes, cells were washed with PBS and incubated with DMEM serum free for 1 h at 37°C. Intracellular ROS formation was measured under a fluorescence microscope (Zeiss Axio Imager M1). Fluorescence images were captured with an AxioVision image recording system computer. Four randomly selected areas with 50–100 cells in each were analyzed and the values obtained are expressed as fold increases in hydrogen peroxide and superoxide anion versus untreated cells.

#### Determination of tert-butyl hydroperoxide-induced intracellular ROS formation

As above, the intracellular hydrogen peroxide and superoxide anion formation induced by tert-butyl hydroperoxide (t-BuOOH) at SH-SY5Y cell level was determined using, respectively, the probes DCFH-DA and DHE. Briefly, SH-SY5Y cells were cultured in 96-well microtiter plates at 3 × 10^4^ cells/well for 24 h. The medium was then removed and the cells were treated for 3 h with various concentrations of compounds (3–30 μmol/L) at 37°C. After treatment, the cells were washed with PBS and then incubated with DCFH-DA (5 μmol/L) or DHE (10 μmol/L) in PBS at 37°C in 5% CO_2_ for 30 min. After removal of the probes and further washing, the cells were incubated with t-BuOOH (50 μmol/L) in PBS for 30 min. At the end of incubation, the green or red fluorescence of the cells from each well was measured with a spectrofluorometer (TECAN®, GENios). The results are expressed as fold increases in intracellular ROS evoked by exposure to t-BuOOH.

#### Determination of total antioxidant activity in membrane and cytosolic fractions

To determine whether AP2469 exerts its antioxidant activity mainly in the membrane or cytosolic fraction, TAA was measured on both the cytosolic and membrane enriched fractions as previously reported (Tarozzi et al. 2012). Briefly, SH-SY5Y cells were cultured in cultures dishes at 4 × 10^6^ cells/dish for 24 h. After incubation, the SH-SY5Y cells were treated for 2 h with various concentrations of AP2469 (3–30 μmol/L). The SH-SY5Y cells were then washed three times with cold PBS, collected in 1 mL of PBS and centrifuged for 10 min at 10,000 rpm at 4°C. The supernatant obtained was removed and the cells were washed with 1 mL of PBS. This was repeated twice more, and the pellet was finally reconstituted in 600 μL of 0.05% Triton X-100. Cells were then homogenized and allowed to stand at 4°C for 30 min. Cytosolic and membrane enriched fractions were subsequently separated by centrifugation at 14,000 rpm for 15 min at 4°C. TAA in cell fractions was determined by the decoloration of the radical cation of 2,2′-azinobis-(3-ethylbenzothiazoline-6-sulfonic acid) (ABTS^•+^), in terms of quenching of absorbance at 740 nm. Values obtained for each sample were compared with the concentration–response curve of a standard antioxidant, such as Trolox (a water-soluble vitamin E analogue), and the results are expressed as μmol of Trolox Equivalent Antioxidant Activity per mg of protein (TEAA μmol/mg protein).

#### Determination of A*β*_1-42_ oligomer-induced nitric oxide and tumor necrosis factor alpha release

THP-1 cells were seeded in 96-well plates at 5 × 10^4^ cells/well for 24 h. At the end of incubation, the medium was changed with a fresh one with AP2469 30 μmol/L for 3 h. The cells were then washed with PBS and incubated for a further 12 h with A*β*_1-42_ oligomer (5 μmol/L). The content of each well was then removed and centrifuged at 2500*g* for 10 min, and the supernatant was frozen at −20°C for the subsequent analysis of released NO and tumor necrosis factor alpha (TNF*α*). To determine the NO levels, 100 μL of supernatant was incubated with 100 μL of Griess reagent (Sigma Aldrich) in the dark at room temperature for 15 min. The intensity of the color that developed was then measured at 540 nm using a spectrophotometer (TECAN®, GENios). Values are reported as percent increases in treated cells versus untreated cells. The TNF*α* levels in 100 μL of supernatant were determined by Ray Bio® ELISA kit according to the manufacturer's instructions (Ray Biotech, Inc., GA). The concentration of TNF*α* levels was calculated from a TNF*α* standard curve standard of 25–6000 pg/mL and the values are reported as percent increases in treated cells versus untreated cells.

### Statistical analysis

Data are reported as mean ± SD of at least three independent experiments. Statistical analysis was performed using one-way ANOVA with Dunnett post hoc test and Student's t-test, as appropriate. Differences were considered significant at *P* < 0.05. Analyses were performed using GraphPad Prism 4.0 software.

## Results

### Effects of AP2469 on cholinesterase and BACE1 activity, and A*β*_1-42_ aggregation

The substitution of the 5,6-dimethoxycoumarin nucleus of AP2238 and AP2243 with the catecholic one reduced the anti-AChE activity by two orders of magnitude (Table 1).

**Table 1:** Inhibitory activities on human AChE and BuChE, BACE1, A*β*1-42 self-aggregation, A*β*1-42 oligomer cytotoxicity of the studied compounds.

Compounds	IC50 μmol/L ± SEM^1^					
	hAChE	hBuChE	BACE1	A*β*1-42 self-aggregation	A*β*1-42 oligomer toxicity	
					SH-SY5Y cells	THP-1 cells
AP2238	0.044 ± 0.006^2^	48.9 ± 3.7^2^	−	≫50^3^	–	–
AP2243	0.018 ± 0.003^4^	118 ± 16^3^	0.24 ± 0.03	≫50^3^	≫30^3^	≫30^3^
AP2469	8.60 ± 0.21	124 ± 13	6.49 ± 0.31	21.7 ± 3.4	7.50 ± 0.72	8.18 ± 0.51
Galantamine	2.01 ± 0.15	20.7 ± 1.5	>5^3^,^5^	≫50^3^	7.99 ± 0.91	≫30^3^

1 Concentration of compound resulting in 50% inhibition of human AChE, BuChE, BACE1 activity, A*β*1-42 self-aggregation, and A*β*1-42 oligomer cytotoxicity.

2 From Piazzi et al. 2003.

3 Not active at the highest tested concentration (50 μmol/L, 30 μmol/L or 5 μmol/L).

4 From Piazzi et al. 2007.

5 From Mancini et al. 2007.

Regarding BACE1 inhibition, AP2469 showed an IC_50_ value of 6.5 μmol/L (Table 1).

As previously reported, AP2238 and AP2243 act as weak inhibitors of hAChE-induced amyloid aggregation (35% and 38%, respectively, at 100 *μ*mol/L concentration, Piazzi et al. 2003, 2007), while they do not show any significant inhibitory activity against amyloid self-aggregation. On the other hand, co-incubation of A*β*_1-42_ with increasing concentrations of AP2469 led to a concentration-dependent reduction in amyloid fibrillization as demonstrated by a ThT-based assay (Table 1).

### Effects of AP2469 on A*β*_1-42_ oligomer- and A*β*_25-35_-induced neurotoxicity

**Figure 2:**
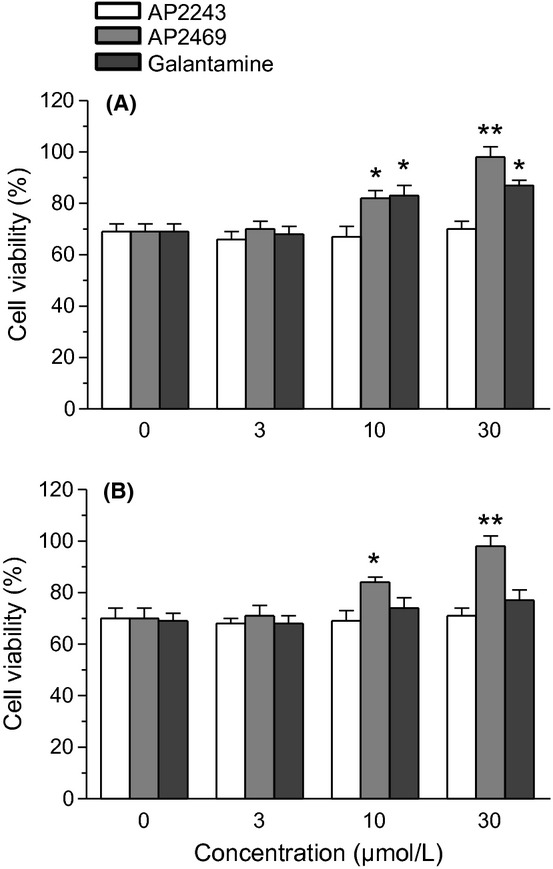
Effects of compounds AP2469, AP2243 and galantamine on A*β*_1-42_ oligomer-induced cytotoxicity in SH-SY5Y (A) and THP-1 (B) cells. The cell viability in SH-SY5Y and THP-1 cells was determined by MTT assay (as described in the Materials and Methods section), after 3 h of incubation with A*β*_1-42_ oligomers (5 μmol/L) in the presence or absence of various concentrations of compounds (1–30 μmol/L). The results are expressed as a percentage of control cells and the values are reported as mean ± SD of three independent experiments (*P < 0.05, **P < 0.01 vs. untreated cells at ANOVA with Dunnett post hoc test).

**Figure 3:**
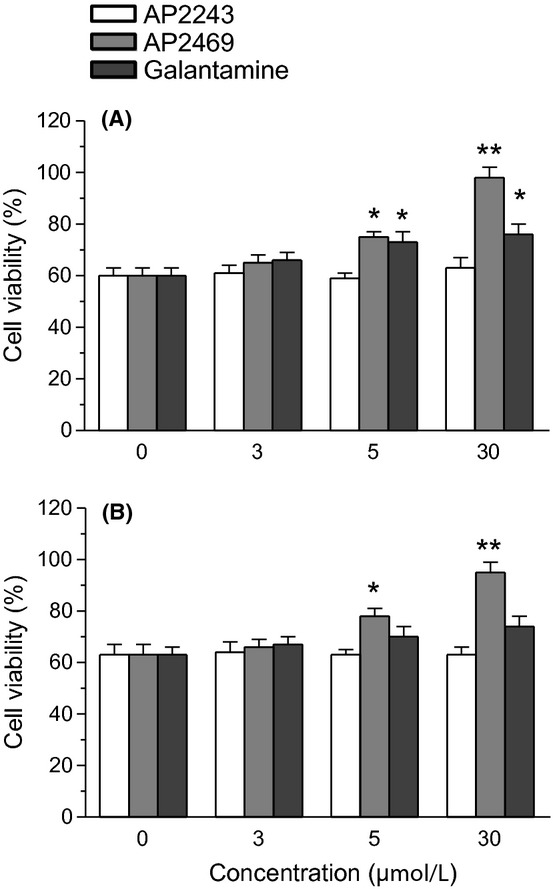
Effects of compounds AP2469, AP2243 and galantamine on A*β*_25-35_-induced cytotoxicity in SH-SY5Y (A) and THP-1 (B) cells. The cell viability in SH-SY5Y and THP-1 cells was determined by MTT assay (as described in the Materials and Methods section), after 3 h of incubation with A*β*_25-35_ (5 μmol/L) in the presence or absence of various concentrations of compounds (1–30 μmol/L). The results are expressed as a percentage of control cells and the values are reported as mean ± SD of three independent experiments (*P < 0.05, **P < 0.01 vs. untreated cells at ANOVA with Dunnett post hoc test).

The ability of AP2469 to interfere with the aggregation processes of A*β*_1-42_ makes this compound eligible for further neuroprotection experiments with human neuronal SH-SY5Y and monocytic THP-1 cell cultures. The SH-SY5Y and THP-1 cells were treated for 3 h with various concentrations, not associated with toxicity, of AP2469, AP2243, and galantamine (1–30 μmol/L) in the presence or absence of A*β*_1-42_ oligomers (5 μmol/L). The same experiments were also performed with A*β*_25-35_ (5 μmol/L), a short peptide generated by proteolysis of A*β*_1-40_ with neurotoxic and aggregation properties similar to A*β*_1-42_ (Tarozzi et al. 2010a,b). Cell toxicity induced by both A*β*_1-42_ oligomers and A*β*_25-35_, in terms of mitochondrial activity loss, was then evaluated by the reduction in MTT to formazan. The treatment of SH-SY5Y cells with AP2469, galantamine, but not AP2243, led to a decrease in cytotoxicity elicited by A*β*_1-42_ oligomers (Table 1). As shown in Figure 2A, the observed decrease in toxicity was dose-dependent and significant for 10 μmol/L and 30 μmol/L of both AP2469 and galantamine. In particular, AP2469 showed a higher neuroprotective effect than galantamine at 30 μmol/L (toxicity inhibition, 93% vs. 58%). In contrast to AP2243 and galantamine, AP2469 also showed the ability to inhibit the A*β*_1-42_ oligomer-induced cytotoxicity in THP-1 cells at 10 μmol/L and 30 μmol/L (Fig. 2B). Similar neuroprotective effects of compounds studied were recorded against A*β*_25-35_-induced toxicity in both SH-SY5Y and THP-1 cells (Fig. 3A and B).

### Effects of AP2469 on A*β*_1-42_ oligomer-binding to plasma membrane and subsequent intracellular ROS formation

**Figure 4:**
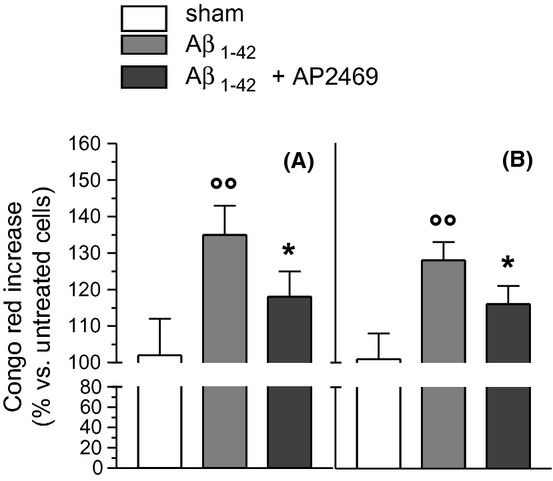
Effects of compounds AP2469 on the binding of A*β*_1-42_ oligomers with SH-SY5Y (A) and THP-1 (B) cells. SH-SY5Y and THP-1 cells were treated with A*β*_1-42_ oligomers (5 μmol/L) for 30 min in the presence or absence of compound AP2469 (30 μmol/L). At the end of incubation, the A*β*_1-42_ oligomer binding to plasma membrane was determined by CR assay (as described in the Materials and Methods section) and the results are expressed as a percentage of control cells. The values are reported as mean ± SD of three independent experiments (°°*P* < 0.01 vs. untreated cells and *P < 0.05 vs. treated cells with A*β*_1-42_ oligomers at Student's *t*-test).

The binding of A*β*_1-42_ oligomers (5 μmol/L) with both SH-SY5Y and THP-1 cells obtained in 30 min was significantly reduced by cotreatment of AP2469, as determined by the CR assay (Fig. 4). In the same experimental conditions, the cotreatment with AP2243 did not modify the binding of A*β*_1-42_ oligomers with both cell types (data not shown).

### Antioxidant activity of AP2469

**Figure 5:**
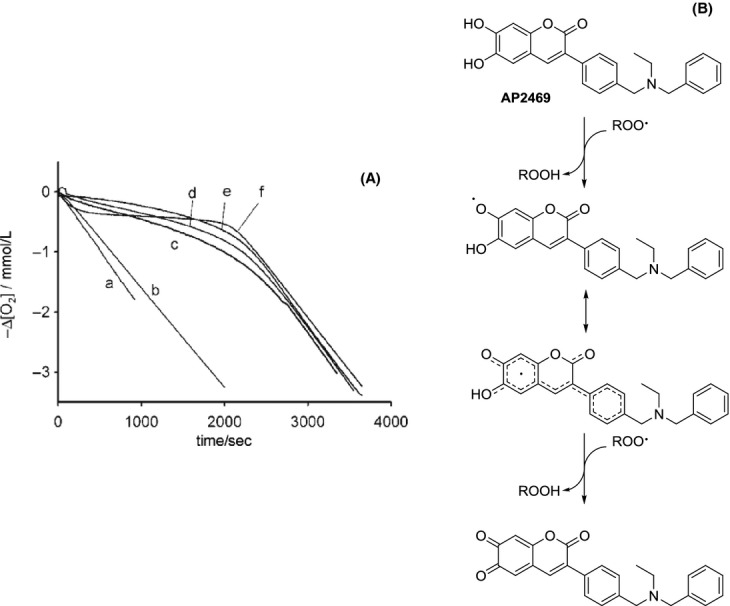
(A) Effects of compounds AP2469 and AP2243 on O_2_ consumption induced by reaction with AIBN and styrene. Oxygen consumption observed during the AIBN (0.05 mol/L)-initiated autoxidation of styrene (4.3 mol/L) at 30°C in chlorobenzene without inhibitors (trace a) or in the presence of 6.5 μmol/L of: AP2243 (b); catechol (c); quercetin (d), AP2469 (e); PMHC (f). (B) Proposed antioxidant action of AP2469.

We evaluated the ability of AP2469 and AP2243 to counteract the peroxyl radicals by EPR spectroscopy. The results clearly showed that the presence of the di-hydroxy moiety dramatically increased the radical trapping ability of AP2469 compared to AP2243 (Table 2). In addition, these results are confirmed by a decrease in O_2_ consumption induced by reaction with AIBN and styrene in the presence of AP2469 (Fig. 5A).

**Table 2:** Intrinsic antioxidant activity of AP2469, AP2243, and of other reference phenolic antioxidants.

Compounds	*k*_*ROO*·_ (mol/L^−1^sec^−1^)^1^	*n*^2^
AP2469	(1.6 ± 0.2) × 106	1.9 ± 0.2
AP2243	<103	–3
Catechol	(5.5 ± 0.5) × 105	2.0 ± 0.2
Quercetin	(5.6 ± 0.5) × 105	2.1 ± 0.2

1 Rate constant for the reaction with peroxyl radicals.

2 Number of radicals trapped by each antioxidant molecule.

3 Not determined.

The rate of reaction of AP2469 with peroxyl radicals was about threefold greater than that recorded for catechol or quercetin under identical experimental settings, indicating that AP2469 forms a phenoxyl (ortho-semiquinone) radical stabilized by resonance on the conjugated double bonds of the stilbene skeleton (Fig. 5B). The number of peroxyl radicals trapped by each AP2469 molecule (the stoichiometric factor, n) was approximately two, similar to that of other catecholic antioxidants, which are known to be oxidized to the corresponding ortho-quinone (Amorati et al. 2006; Valgimigli and Pratt 2012).

**Figure 6:**
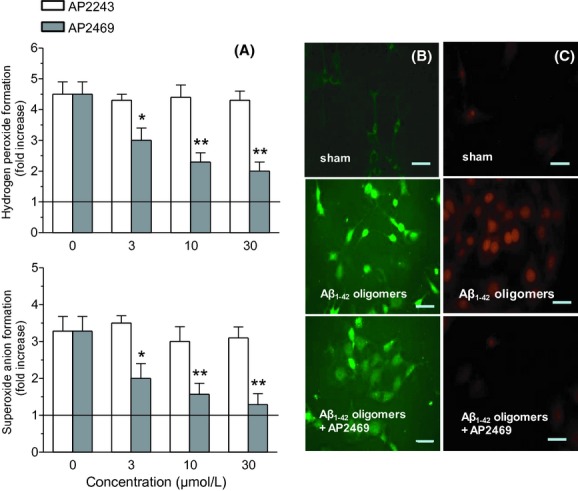
Effects of compounds AP2469 and AP2243 on A*β*_1-42_ oligomer-induced ROS formation in SH-SY5Y cells. (A) SH-SY5Y cells were treated for 3 h with various concentrations of compound AP2469 (3–30 μmol/L) prior to the treatment of 3 h with A*β*_1-42_ oligomer (5 μmol/L). At the end of incubation, hydrogen peroxide and superoxide anion formation was determined using, respectively, the probe, DCFH-DA and DHE, as described in the Materials and Methods section. Four randomly selected areas with 50–100 cells in each were analyzed under a fluorescence microscope and the values obtained are expressed as fold increases in ROS formation induced by exposure to A*β*_1-42_ oligomers. The values are shown as mean ± SD of three independent experiments (*P < 0.05, **P < 0.01 vs. untreated cells at Student's *t*-test). Representative images of hydrogen peroxide (B) and superoxide anion (C) formation. Scale bars: 100 μm.

**Figure 7:**
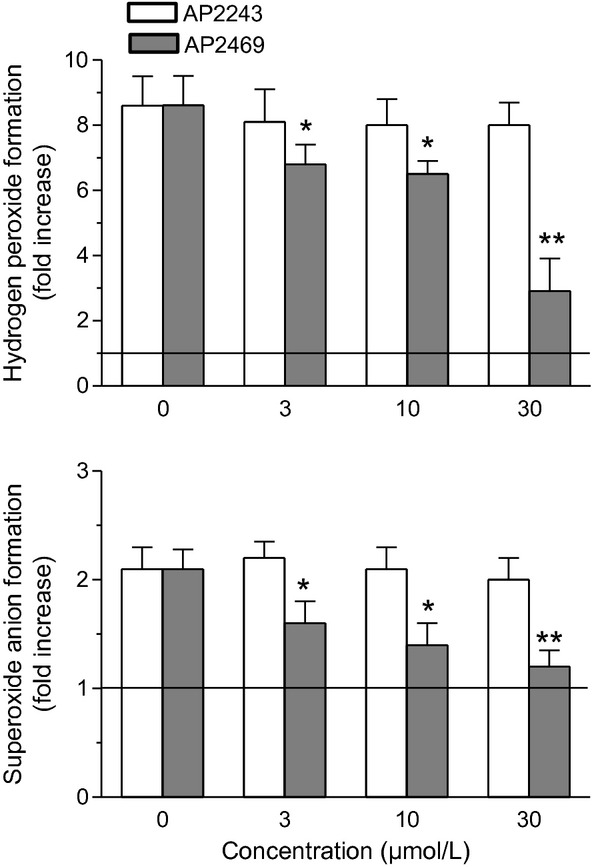
Effects of compounds AP2469 and AP2243 on *t*-BuOOH-induced ROS formation in SH-SY5Y cells. SH-SY5Y cells were treated for 3 h with various concentrations of AP2469 and AP2243 (3–30 μmol/L) prior to the treatment of 30 min with *t-*BuOOH (50 μmol/L). At the end of incubation, hydrogen peroxide and superoxide anion formation was determined using, respectively, the probe, DCFH-DA and DHE (as described in the Materials and Methods section). The results are expressed as fold increases in ROS formation induced by exposure to *t*-BuOOH and the values are reported as mean ± SD of three independent experiments (*P < 0.05, **P < 0.01 vs. untreated cells at ANOVA with Dunnett post hoc test).

Then we also evaluated the ability of AP2469 and AP2243 to counteract intracellular ROS formation, such as hydrogen peroxide and superoxide anion, evoked by A*β*_1-42_ oligomers (5 μmol/L) in SH-SY5Y cells using DCFH-DA and DHE assays, respectively. The ROS formation has been directly related to membrane and cytoplasm perturbation by soluble oligomers (Glabe 2006; Rauk 2008). As shown in Figure 6, AP2469 significantly inhibited the A*β*_1-42_ oligomer-induced hydrogen peroxide and superoxide anion formation at all used concentrations (3–30 μmol/L). In parallel, we also investigated the antioxidant activity of AP2469 and AP2243 against the t-BuOOH-induced ROS formation, in SH-SY5Y using same assays. t-BuOOH is an organic peroxide that generates a pattern of ROS similar to that involved in the oxidative stress induced by A*β* Rauk 2008). Remarkably, pretreatment of both the cell types with AP2469, but not AP2243, at all selected concentrations (3–30 μmol/L) significantly reduced the ROS formation induced by t-BuOOH at 50 μmol/L (Figure 7).

**Figure 8:**
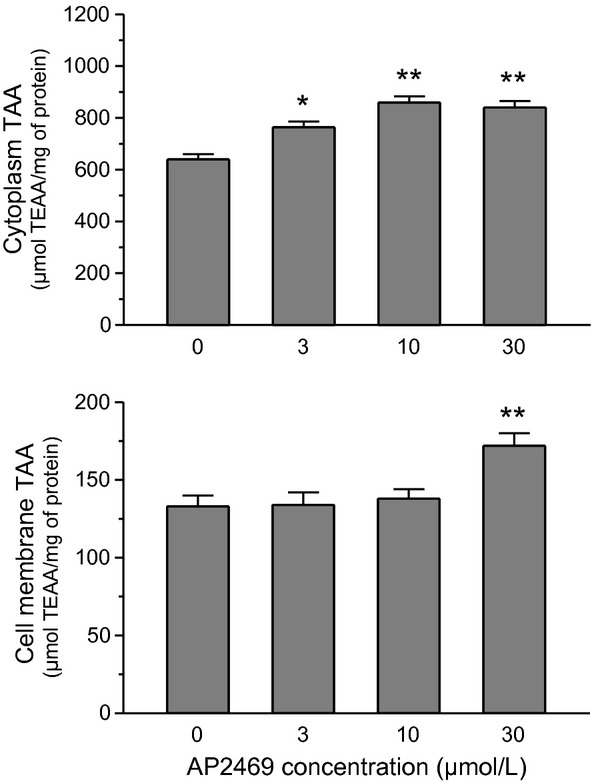
Total antioxidant activity of membrane and cytosolic fraction from SH-SY5Y cells treated with AP2469. SH-SY5Y cells were treated for 2 h with various concentrations of AP2469 (3–30 μmol/L), and cytosolic and membrane fractions were separated. Both cellular fractions were submitted to the ABTS radical cation decolorization assay (as described in the Materials and Methods section). The results are expressed as μmol of TEAA per mg of protein and the values are shown as mean ± SD of three independent experiments (*P < 0.05, **P < 0.01 vs. untreated cells at ANOVA with Dunnett post hoc test).

In order to better evaluate the ability of AP2469 to exert its antioxidant activity in SH-SY5Y cells, and to obtain an indirect evaluation of the cellular bioavailability of AP2469, we measured the total antioxidant activity (TAA) of cytosolic and membrane enriched fractions of SH-SY5Y cells treated with the compound. As reported in Figure 8, both the membrane and cytosolic fractions obtained from SH-SY5Y cells treated for 2 h with AP2469 showed a significant increase in TAA in comparison to untreated cells. A significant TAA increase was found at cytosolic level with all AP2469 tested concentrations (3–30 μmol/L), while, at membrane levels, the TAA increase was significant only at the highest tested concentration (30 μmol/L).

### Effects of AP2469 on A*β*_1-42_ oligomer-induced NO and TNF*α* release

**Figure 9:**
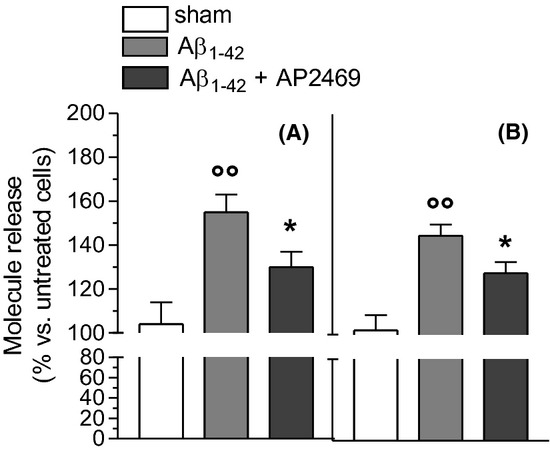
Compound AP2469 inhibits A*β*_1-42_ oligomer-induced NO (A) and TNF*α* (B) release in THP-1 cells. THP-1 cells were treated for 3 h with compound AP2469 (30 μmol/L) prior to the treatment for 12 h with A*β*_1-42_ oligomer (5 μmol/L). At the end of incubation, NO and IL-1 release was estimated using the Griess reagent and Ray Bio® ELISA kit, respectively (as described in the Materials and Methods section). The results are expressed as a percentage of control cells and the values are reported as mean ± SD of three independent experiments (°°*P* < 0.01 vs. untreated cells and *P < 0.05 vs. treated cells with A*β*_1-42_ oligomers at Student's *t*-test).

A*β*_1-42_ oligomers are known to increase levels of inducible nitric oxide synthase (iNOS) in microglial cells with gial activation and release of proinflammatory cytokines (Dheen et al. 2005; Ajit et al. 2009). As the induction of iNOS is associated with increased production of NO, we examined the effects of AP2469 30 μmol/L on production of NO using Griess reagent. We found that cotreatment of THP-1 cells with AP2469 reduced A*β*_1-42_ oligomer-induced NO release (Fig. 9A). In parallel, the same treatment inhibited the TNF*α* release from THP-1 cells (Fig. 9B).

## Discussion

AD's multifactorial origin has stimulated the recent therapeutic approach in which drug candidates are designed to possess different pharmacological properties and act on multiple targets.

In light of the development of a new multifunctional compound with a broader spectrum of action, we hypothesized that the simple structural modification of the dimethoxycoumarin nucleus of AP2243 to give a catechol-bearing derivative could lead to a new compound endowed with both anticholinesterase and antiaggregating properties, together with antioxidant, neuroprotective, anti-inflammatory activity at neuronal and microglial cell level.

The substitution of the 5,6-dimethoxycoumarin nucleus of AP2238 and AP2243 with the catecholic one reduced the anti-AChE activity by two orders of magnitude, thus underlying the importance of the dimethoxycoumarin fragment for optimal anticholinesterase activity. Nevertheless, in light of a balanced inhibition profile, a partial loss of the inhibitory potency against one target is acceptable if other pharmacological properties are gained and can be simultaneously exerted. Moreover, an IC_50_ value in the micromolar range, as for AP2469, is likely suitable to elevate the cholinergic tone as demonstrated by the clinical use of the natural alkaloid galantamine, an AChE-selective inhibitor with a micromolar inhibitory potency. Like galantamine, AP2469 is a selective AChE inhibitor even if selectivity is reduced by one order of magnitude with respect to AP2243 and AP2238.

On the basis of the inhibitory activity of quercetin against BACE1 activity in both a cell-free system and in neuronal cells (Shimmyo et al. 2008) the ability of AP2469 was investigated. Regarding BACE1 inhibition, AP2469 showed a potency of one order of magnitude lower than that of AP2243, but exhibited a BACE1 inhibitory activity very similar to that found for other nonpeptidic inhibitors such as the noncompetitive BACE1 inhibitor bis(7)-tacrine (7.5 μmol/L, Fu et al. 2008) or the catechol derivative quercetin (5.4 μmol/L, Shimmyo et al. 2008).

As previously reported, AP2238 and AP2243 act as weak inhibitors of hAChE-induced amyloid aggregation (Piazzi et al. 2003, 2007) while they do not show any significant inhibitory activity against amyloid self-aggregation. Due to this weak activity profile of both previously published compounds and the lower inhibitory activity of AP2469 on hAChE, AChE-induced amyloid aggregation was not considered a potential target of action for AP2469.

On the other hand, AP2469 is able to interfere with A*β*_42_ oligomerization in a concentration-dependent manner.

The ability of AP2469 to interfere with the aggregation processes of A*β*_1-42_ makes this compound eligible for further neuroprotection experiments with SH-SY5Y and THP-1 cell cultures. The treatment of SH-SY5Y cells with AP2469 and galantamine, but not AP2243, led to a dose-dependent decrease in cytotoxicity elicited by both A*β*_1-42_ oligomers and A*β*_25-35_ peptide. Similarly, treatment with AP2469, but not galantamine or AP2243, showed significant inhibitor effects on A*β*-induced toxicity in THP-1 cells.

The AP2469 neuroprotective effect findings prompted us to evaluate its ability to prevent the binding between A*β*_1-42_ oligomer and the plasma membrane surface, an action that triggers irreversible membrane alterations and initiates a sequence of pathological events leading to cell dysfunction and death. The binding of A*β*_1-42_ oligomers with both SH-SY5Y and THP-1 cells was significantly reduced by cotreatment of AP2469. In the same experimental conditions, the cotreatment with AP2243 did not modify the binding of A*β*_1-42_ oligomers with both cell types. These results show that AP2469 and galantamine afford cytoprotection through mechanisms that are likely unrelated to AChE inhibition (e.g., AP2243 inhibits the AChE activity, but not A*β*-induced cytotoxicity). The ability of galantamine to exert the cytoprotective effects in SH-SY5Y cells is confirmed by other studies that linked these effects with the agonist action of galantamine on nicotinic receptors (Arias et al. 2005). In contrast to galantamine, the cytoprotective effects of AP2469 in both SH-SY5Y and THP-1 cells could be attributed to inhibitory effects on early pathological events leading to cell death, such as A*β*_1-42_ oligomer-binding to plasma membrane and subsequent toxicity. Interestingly, the ability of AP2469 to exert the neuroprotective effects observed in neuronal SH-SY5Y cells also in microglial-like THP-1 cells suggests that this compound could preserve the neuroprotective effects of microglial activation, such as phagocytosis of dead neurons and clearance of debris (Polazzi and Monti 2010).

Regarding this evidence, it is plausible that AP2469 interacts directly with specific amino acids present in the A*β* peptide. In contrast to AP2243, AP2469 has a di-hydroxy moiety which could form hydrogen bonds with acceptor groups of amino acid residues in A*β* peptide (i.e., Ile31, Ile32 and Met35) that are critical for its aggregation and subsequent cytotoxicity (Pike et al. 1995; Clementi et al. 2005).

Finally, the promising results obtained on THP-1 cell plasma membranes prompted us to evaluate the anti-inflammatory effects of AP2469 in terms of prevention of glial activation. A*β*_1-42_ oligomers are known to increase levels of inducible nitric oxide synthase (iNOS) in microglial cells with glial activation and release of proinflammatory cytokines (Dheen et al. 2005; Ajit et al. 2009). As the induction of iNOS is associated with increased production of NO, we next examined the effects of AP2469 on production of NO. We found that cotreatment of THP-1 cells with AP2469 reduced A*β*_1-42_ oligomer-induced NO release. In parallel, the same treatment inhibited the TNF*α* release from THP-1 cells.

The ability of AP2469 to modulate the THP-1 cells' proinflammatory effects induced by A*β*_1-42_ oligomers is particularly significant, as microglial cells can play an important role in the activation of neuroinflammation, which has been linked to the neuronal death associated with AD (Glass et al. 2010).

Integrated studies of antioxidant activity clearly showed that the presence of the catechol moiety also dramatically increased the radical trapping ability of AP2469 compared to AP2243, and reduced the intracellular formation of hydrogen peroxide and superoxide anion induced by both A*β*_1-42_ oligomers and t-BuOOH in SH-SY5Y cells. Taken together, these data highlight the very good chain-breaking antioxidant performance of AP2469 and its potential in counteracting oxidative damage at neuron level involved in AD pathogenesis and progression. In this regard, recent studies have suggested that A*β*_1-42_ as small oligomers can insert themselves into the lipid bilayer and initiate lipid peroxidation and, consequently, oxidative damage to proteins and other biomolecules (Butterfield et al. 2001; Butterfield and Sultana 2011). Therefore, it is also probable that the antioxidant effects observed after pretreatment of SH-SY5Y with AP2469 is ascribed to its ability: (1) to spread into the membrane and to form a barrier on the membrane through the formation of hydrogen bonds with the polar head groups at the lipid–water interface of the plasma membrane and protect it from external prooxidant aggressors including A*β* peptides; (2) to prevent the formation of sulfuranyl radicals from one electron oxidation of Met35 present in A*β*_1-42_, which can then initiate the process of lipid peroxidation (Butterfield and Sultana 2011); (3) to enter into the cytoplasm and to exert the antioxidant activity at this level. The ability of AP2469 to cross the cytoplasmatic membrane and to reach the cytoplasm of SH-SY5Y cells suggests a favorable pharmacokinetic profile of this molecule. As final targets are located in the CNS, the possibility for AP2469 to penetrate the blood–brain barrier (BBB) was also estimated by calculating physico-chemical properties, known to influence BBB penetration. In particular, with a log *P* value of 4.60, AP2649 is significantly more lipophilic than catechol (log *P* = 1.09) and quercetin (log *P* = 0.35). With a number of hydrogen bond donors ≤3 (n. H-bond donor = 2), hydrogen bond acceptor largely ≤7 (n. H-bond acceptor = 3), and molecular weight close to 400 g/mol (401.45 g/mol), the physico-chemical property profile of AP2469 is in compliance with Lipinski's and Wenlock's guidelines for good passive CNS penetration (Wenlock et al. 2003; Pajouhesh and Lenz 2005).

In conclusion, starting from the first dual binding site AChE inhibitor AP2238, with a simple structural modification, we were able to obtain a multi-target ligand. Indeed, we followed the new medicinal chemistry paradigm which states that, in order to significantly modify the progression of a multifactorial disease such as AD, the modulation of the activity of a single-protein target may not be appropriate. Therefore, with the introduction of a catechol moiety, we obtained AP2469 that possesses anti-AChE and anti-BACE1 activities, and shows an enlarged activity profile able to interact with other key targets for AD. Specifically, the combined neuroprotective and anti-inflammatory properties and its potential in counteracting oxidative damage in both neuron and microglial cells make AP2469 a potential drug candidate able to modify the course of the disease.

## Abbreviations

AChE: acetylcholinesterase
AIBN: azobisisobutyronitrile
A*β*: *β*-amyloid peptide
BACE1: beta-site amyloid precursor protein cleaving enzyme 1
BuChE: butyrylcholinesterase
CR: Congo red
DCFH-DA: 2′,7′-dichlorodihydrofluorescein diacetate
DHE: dihydroethidium
EPR: electron paramagnetic resonance
MTT: 3-(4,5-dimethyl-2-thiazolyl)-2,5-diphenyl-2H-tetrazolium bromide
NO: nitric oxide
ROS: reactive oxygen species
TAA: total antioxidant activity
t-BuOOH: tert-butyl hydroperoxide
ThT: thioflavin T
TNF: tumor necrosis factor

## References

[b1] Ajit D, Udan ML, Paranjape G, Nichols MR (2009). Amyloid-beta(1-42) fibrillar precursors are optimal for inducing tumor necrosis factor-alpha production in the THP-1 human monocytic cell line. Biochemistry.

[b2] Amorati R, Valgimigli L (2012). Modulation of the antioxidant activity of phenols by non-covalent interactions. Org Biomol Chem.

[b3] Amorati R, Pedulli GF, Valgimigli L, Attanasi OA, Filippone P, Fiorucci C (2001). Absolute rate constants for the reaction of peroxyl radicals with cardanol derivatives. J Chem Soc Perkin Trans.

[b4] Amorati R, Pedulli GF, Cabrini L, Zambonin L, Landi L (2006). Solvent and pH effects on the antioxidant activity of caffeic and other phenolic acids. J Agric Food Chem.

[b5] Amorati R, Pedulli GF, Pratt DA, Valgimigli L (2010). TEMPO reacts with oxygen-centered radicals under acidic conditions. Chem Commun.

[b6] Amorati R, Pedulli GF, Valgimigli L (2011). Kinetic and thermodynamic aspects of the chain-breaking antioxidant activity of ascorbic acid derivatives in non-aqueous media. Org Biomol Chem.

[b7] Arias E, Gallego-Sandín S, Villarroya M, García AG, López MG (2005). Unequal neuroprotection afforded by the acetylcholinesterase inhibitors galantamine, donepezil, and rivastigmine in SH-SY5Y neuroblastoma cells: role of nicotinic receptors. J Pharmacol Exp Ther.

[b8] Bartolini M, Bertucci C, Bolognesi ML, Cavalli A, Melchiorre C, Andrisano V (2007). Insight into the kinetic of amyloid beta (1-42) peptide self-aggregation: elucidation of inhibitors' mechanism of action. ChemBioChem.

[b9] Block ML, Zecca L, Hong JS (2007). Microglia-mediated neurotoxicity: uncovering the molecular mechanisms. Nat Rev Neurosci.

[b10] Bobba A, Petragallo VA, Marra JS (2010). Alzheimer's proteins, oxidative stress, and mitochondrial dysfunction interplay in a neuronal model of Alzheimer's disease. Int J Alzheimers Dis.

[b11] Burton GW, Doba T, Gabe EJ, Hughes L, Lee FL, Prasad L (1985). Autoxidation of biological molecules. 4. Maximizing the antioxidant activity of phenols. J Am Chem Soc.

[b12] Butterfield DA, Sultana R (2011). Methionine-35 of aβ(1-42): importance for oxidative stress in Alzheimer disease. J Amino Acids.

[b13] Butterfield DA, Drake J, Pocernich C, Castegna A (2001). Evidence of oxidative damage in Alzheimer's disease brain: central role for amyloid beta-peptide. Trends Mol Med.

[b14] Citron M (2002). Beta-secretase as a target for the treatment of Alzheimer's disease. J Neurosci Res.

[b15] Clementi ME, Marini S, Coletta M, Orsini F, Giardina B, Misiti F (2005). Abeta(31-35) and Abeta(25-35) fragments of amyloid beta-protein induce cellular death through apoptotic signals: role of the redox state of methionine-35. FEBS Lett.

[b16] Dheen ST, Jun Y, Yan Z, Tay SS, Ling EA (2005). Retinoic acid inhibits expression of TNF-alpha and iNOS in activated rat microglia. Glia.

[b17] Di Giovanni S, Eleuteri S, Paleologou KE, Yin G, Zweckstetter M, Carrupt PA (2010). Entacapone and tolcapone, two catechol O-methyltransferase inhibitors, block fibril formation of alpha-synuclein and beta-amyloid and protect against amyloid-induced toxicity. J Biol Chem.

[b18] Ellman GL, Courtney KD, Andres V, Featherstone RM (1961). A new rapid colorimetric determination of acetylcholinesterase activity. Biochem Pharmacol.

[b19] Estrada LD, Soto C (2007). Disrupting *β*-Amyloid aggregation for Alzheimer's disease treatment. Curr Topics Med Chem.

[b201] Fine RE (1999). The biochemistry of Alzheimer disease. Alzheimer Dis Assoc Disord.

[b20] Fu H, Li W, Luo J, Lee NT, Li M, Tsim KW (2008). Promising anti-Alzheimer's dimer bis(7)-tacrine reduces beta-amyloid generation by directly inhibiting BACE-1 activity. Biochem Biophys Res Commun.

[b21] Glabe CG (2006). Common mechanisms of amyloid oligomer pathogenesis in degenerative disease. Neurobiol Aging.

[b22] Glass CK, Saijo K, Winner B, Marchetto MC, Gage FH (2010). Mechanisms underlying inflammation in neurodegeneration. Cell.

[b23] Guo T, Hobbs DW (2006). Development of BACE1 inhibitors for Alzheimer's disease. Curr Med Chem.

[b24] Hardy J, Selkoe DJ (2002). The amyloid hypothesis of Alzheimer's disease: progress and problems on the road to therapeutics. Science.

[b25] Hills ID, Vacca JP (2007). Progress toward a practical BACE-1 inhibitor. Curr Opin Drug Discov Devel.

[b26] Huong VT, Shimanouchi T, Shimauchi N, Yagi H, Umakoshi H, Goto Y (2010). Catechol derivatives inhibit the fibril formation of amyloid-beta peptides. J Biosci Bioeng.

[b27] Klegeris A, McGeer PL (2000). Interaction of various intracellular signaling mechanisms involved in mononuclear phagocyte toxicity toward neuronal cells. J Leukoc Biol.

[b28] Kowall NW (1999). Alzheimer disease 1999: a status report. Alzheimer Dis Assoc Disord.

[b29] LeVine H (1993). Thioflavine T interaction with synthetic Alzheimer's disease beta-amyloid peptides: detection of amyloid aggregation in solution. Protein Sci.

[b30] Maezawa I, Hong HS, Wu HC, Battina SK, Rana S, Iwamoto T (2006). A novel tricyclic pyrone compound ameliorates cell death associated with intracellular amyloid-beta oligomeric complexes. J Neurochem.

[b31] Mancini F, Naldi M, Cavrini V, Andrisano V (2007). Multiwell fluorometric and colorimetric microassays for the evaluation of beta-secretase (BACE-1) inhibitors. Anal Bioanal Chem.

[b32] Naiki H, Higuchi K, Nakakuki K, Takeda T (1991). Kinetic analysis of amyloid fibril polymerization in vitro. Lab Invest.

[b33] Pajouhesh H, Lenz RG (2005). Medicinal chemical properties of successful central nervous system drugs. NeuroRx.

[b34] Piazzi L, Rampa A, Bisi A, Gobbi S, Belluti F, Cavalli A (2003). ω-3-(4-{[Benzyl(Methyl)Amino]-Methyl}Phenyl)-6,7-Dimethoxy-2H-2-Chromenone (AP2238) inhibits both acetylcholinesterase and acetylcholinesterase-induced beta-amyloid aggregation a dual function lead for Alzheimer's disease therapy. J Med Chem.

[b35] Piazzi L, Cavalli A, Belluti F, Bisi A, Gobbi S, Rizzo S (2007). Estensive SAR and computational studies of 3-(4-{[Benzyl(methyl)amino]methyl}-phenyl)-6,7-dimethoxy-2H-2-chromenone (AP2238) derivatives. J Med Chem.

[b36] Pike CJ, Walencewicz-Wasserman AJ, Kosmoski J, Cribbs DH, Glabe CG, Cotman CW (1995). Structure-activity analyses of beta-amyloid peptides: contributions of the beta 25-35 region to aggregation and neurotoxicity. J Neurochem.

[b37] Polazzi E, Monti B (2010). Microglia and neuroprotection: from in vitro studies to therapeutic applications. Prog Neurobiol.

[b38] Rauk A (2008). Why is the amyloid beta peptide of Alzheimer's disease neurotoxic?. Dalton Trans.

[b39] Selkoe DJ (1993). Physiological production of the beta-amyloid protein and the mechanism of Alzheimer's disease. Trends Neurosci.

[b40] Selkoe DJ (1994). Normal and abnormal biology of the beta-amyloid precursor protein. Annu Rev Neurosci.

[b41] Shimmyo Y, Kihara T, Akaike A, Niidome T, Sugimoto H (2008). Flavonols and flavones as BACE-1 inhibitors: structure-activity relationship in cell-free, cell-based and in silico studies reveal novel pharmacophore features. Biochim Biophys Acta.

[b42] Talaga P (2001). *β*-Amyloid aggregation inhibitors for the treatment of Alzheimer's disease: dream or reality? *Mini rewiews Med*. Chem.

[b43] Tamagno E, Guglielmotto M, Aragno M, Borghi R, Autelli R, Giliberto L (2008). Oxidative stress activates a positive feedback between the gamma- and beta-secretase cleavages of the beta-amyloid precursor protein. J Neurochem.

[b44] Tarozzi A, Merlicco A, Morroni F, Bolondi C, Di Iorio P, Ciccarelli R (2010a). Guanosine protects human neuroblastoma cells from oxidative stress and toxicity induced by Amyloid-beta peptide oligomers. J Biol Regul Homeost Agents.

[b45] Tarozzi A, Morroni F, Merlicco A, Bolondi C, Teti G, Falconi M (2010b). Neuroprotective effects of cyanidin 3-O-glucopyranoside on amyloid beta (25-35) oligomer-induced toxicity. Neurosci Lett.

[b46] Tarozzi A, Morroni F, Bolondi C, Sita G, Hrelia P, Djemil A (2012). Neuroprotective effects of erucin against 6-hydroxydopamine-induced oxidative damage in a dopaminergic-like neuroblastoma cell line. Int J Mol Sci.

[b47] Valgimigli L, Chatgilialoglu C, Studer A, Pratt DA (2012). Antioxidants in chemistry and biology. Encyclopedia of radicals in chemistry, biology and materials.

[b48] Wenlock MC, Austin RP, Burton P, Davis AM, Leeson PD (2003). A comparison of physicochemical property profiles of development and marketed oral drugs. J Med Chem.

[b49] Yates SL, Burgess LH, Kocsis-Angle J, Antal JM, Dority MD, Embury PB (2000). Amyloid beta and amylin fibrils induce increases in proinflammatory cytokine and chemokine production by THP 1 cells and murine microglia. J Neurochem.

